# Following a Foraging Fish-Finder: Diel Habitat Use of Blainville's Beaked Whales Revealed by Echolocation

**DOI:** 10.1371/journal.pone.0028353

**Published:** 2011-12-07

**Authors:** Patricia Arranz, Natacha Aguilar de Soto, Peter T. Madsen, Alberto Brito, Fernando Bordes, Mark P. Johnson

**Affiliations:** 1 Biodiversidad, Ecología Marina y Conservación (BIOECOMAC), Department of Animal Biology, La Laguna University, Tenerife, Spain; 2 Leigh Marine Laboratory, University of Auckland, Leigh, New Zealand; 3 Section for Zoophysiology, Department of Bioscience, Aarhus University, Aarhus, Denmark; 4 Woods Hole Oceanographic Institution, Woods Hole, Massachusetts, United States of America; 5 Instituto de Ciencias Marinas, Gran Canaria, Spain; 6 Sea Mammal Research Unit, Scottish Oceans Institute, University of St Andrews, St Andrews, Scotland; Institut Pluridisciplinaire Hubert Curien, France

## Abstract

Simultaneous high resolution sampling of predator behavior and habitat characteristics is often difficult to achieve despite its importance in understanding the foraging decisions and habitat use of predators. Here we tap into the biosonar system of Blainville's beaked whales, *Mesoplodon densirostris,* using sound and orientation recording tags to uncover prey-finding cues available to echolocating predators in the deep-sea. Echolocation sounds indicate where whales search and encounter prey, as well as the altitude of whales above the sea-floor and the density of organisms around them, providing a link between foraging activity and the bio-physical environment. Tagged whales (n = 9) hunted exclusively at depth, investing most of their search time either in the lower part of the deep scattering layer (DSL) or near the sea-floor with little diel change. At least 43% (420/974) of recorded prey-capture attempts were performed within the benthic boundary layer despite a wide range of dive depths, and many dives included both meso- and bentho-pelagic foraging. Blainville's beaked whales only initiate searching when already deep in the descent and encounter prey suitable for capture within 2 min of the start of echolocation, suggesting that these whales are accessing prey in reliable vertical strata. Moreover, these prey resources are sufficiently dense to feed the animals in what is effectively four hours of hunting per day enabling a strategy in which long dives to exploit numerous deep-prey with low nutritional value require protracted recovery periods (average 1.5 h) between dives. This apparent searching efficiency maybe aided by inhabiting steep undersea slopes with access to both the DSL and the sea-floor over small spatial scales. Aggregations of prey in these biotopes are located using biosonar-derived landmarks and represent stable and abundant resources for Blainville's beaked whales in the otherwise food-limited deep-ocean.

## Introduction

Foraging animals must locate food resources that are often patchy, and that change in composition and density with time and space [1], [2]. For most terrestrial animals, the challenge of finding food is largely restricted to two spatial dimensions [3], [4] where a series of biotic and abiotic landmarks may aid the location of energy resources [5], [6]. However, for deep sea predators, prey are distributed in a 3-dimensional world of high pressure and often complete darkness that offers fewer or less obvious landmarks. Nonetheless, there are some important concentrating factors in the deep sea. Most biomass below the photic layer concentrates in two vertical strata: the deep scattering layer (DSL) [7] and the benthic boundary layer (BBL) [8]. During the day the DSL is a discrete and dense layer, consisting mostly of small (2–15 cm long) organisms, located at depths between 400 and 800 m, while at night the more active species in the DSL disperse upwards to forage [7], [9], [10]. Species inhabiting the deeper part of the DSL tend to have reduced locomotion and perform limited or no vertical migrations [11], [12] resulting in diel stability of a portion of the DSL. The BBL is considered to extend from the sea-floor to some 200 m altitude above it [13]. It holds most of the biomass in abyssal waters (1000–3500 m depth) and comprises typically species with low locomotory capacity [14].

The DSL and BBL constitute important foraging resources for a variety of oceanic necton including air-breathing top-predators such as marine mammals [15], [16]. However, the abundance of prey within these layers must be balanced against the increased transport and search costs required to find and access them, especially for air breathers with limited dive times [17], [18]. Several species of small marine mammals access DSL organisms when these migrate to shallow depths during the night [15], [16], [19]. This behavior saves air-breathing predators transport costs but halves their potential foraging time by restricting foraging access to night-times only. Other species such as elephant seals and larger toothed whales have developed the ability to perform long and deep breath-hold dives enabling them to forage in the DSL and even the BBL during day and night [20], [21]. Some of these species switch between different prey types and depth layers on a dive-by-dive basis [19], [22]. However, little is known about the governing factors behind such transitions, in large part because of the difficulties involved in sampling both the behavior of deep diving predators and the composition of their bio-physical environment over similar spatial and temporal scales.

A number of studies have related predator movements to biophysical oceanographic features that influence productivity at the scales of kilometres and days to establish the extent of habitats for some species [2], [23]–[25]. However, the temporal and spatial resolutions of such studies are insufficient to resolve specific foraging patterns as defined by local resource distributions [26]. At the other extreme, multi-sensor tags incorporating variously video, sound, and movement sampling can pin-point to the second where and when deep diving predators find prey over short time intervals [27]–[29], but say little about the spatial extent of the targeted prey patches.

Acoustic recording tags (such as the Dtag) [30] are well suited for studying deep-diving echolocating whales and specific acoustic signatures have been identified for when these animals are searching for, and attempting to capture, prey [31], [32]. Comparing the occurrence of these signatures in sperm, pilot and beaked whales foraging in similar habitats, shows that the depths at which prey are encountered vary widely even within individual foraging dives, suggesting that these whales may be accessing a range of prey resources that change in time and space [19], [33], [34]. However, the difficulties involved in relating this detailed foraging behavior back to biotic parameters of the habitat are demonstrated by two recent studies. Hazen *et al.* [35] combined a ship-borne echosounder survey with passive acoustic detections of a deep-diving predator, Blainville's beaked whale (*Mesoplodon densirostris*), to show that this species was more abundant in areas where the backscatter strength of the DSL was large. However, the uniform 400–600 m depth of the DSL in the study site is at odds with the 700–1100 m foraging depths of the same species tagged with acoustic tags in the same area and at the same general time [36]. This apparent contradiction suggests either that the prey sampling methods are too coarse or that there is a more complex ecological relationship between beaked whales and the DSL, requiring simultaneous high resolution sampling of prey abundance and predator behavior. This scale problem is especially acute in areas with high spatial variability such as the steep bathymetric zones around shelf edges, sea-mounts or oceanic islands often favored by deep-diving predators [24], [37].

Here we use a novel source of information providing simultaneous sampling of a predator and its environment to examine the foraging ecology of Blainville's beaked whales. Acoustic tags on this species record both the echolocation sounds produced by tagged whales, including distinctive buzzes indicating prey capture attempts, but also the echoes from the sea surface, sea-floor and organisms in the water column, insonified by these sounds [31], [32]. These data create a unique opportunity to uncover some of the instantaneous cues available to a predator in the deep sea for finding its prey, providing a direct connection between the whale's bio-physical environment, as it encounters it, and its foraging efforts. Using tag recordings from nine Blainville's beaked whales diving in steep bathymetry near an oceanic island, we show that foraging is concentrated around the deep scattering and benthic layers and that whales often switch between meso- and bentho-pelagic foraging within the same dive in a way that is not always evident from the dive profile. We explore the ecological implications of this foraging behavior for prey and habitat selection by this air-breathing top-predator in the deep ocean.

## Methods

### Ethics statement

Animal tagging was approved by the Woods Hole Oceanographic Institutional Animal Care and Use Committee (proposal 7175) and conducted under a permit issued by the Canary Islands Government to N. Aguilar Soto from La Laguna University (Permits # 21/2004, 41/2005, 132/2006, 487/2007, 269/2008, 261/2009).

### Data collection

Field work was performed from 2003 to 2010 off El Hierro, in the Canary Islands, where there is a year-round population of Blainville's beaked whales close to shore. Suction-cup attached digital recorders (Dtag) [30] were used to collect acoustic and movement data. The tags sampled depth and orientation of the whales at 50 Hz and these sensor streams were decimated to 5 Hz for analysis. Acoustic data were sampled from one or two hydrophones at 96 kHz (in 2003) and 192 kHz (in 2004 and onwards) [38]. Data were gathered from 9 individual Blainville's beaked whales in 14 tag deployments. Whales were identified with the aid of photos of their individually-distinctive scar patterns but the difficulty in recognizing individuals at the moment of tagging resulted in four animals being tagged more than once in different years (Table 1). Impact on the whales due to re-tagging is expected to be small given the long inter-tagging intervals (>1 year) and the short-term superficial attachment of the Dtag. To further reduce the potential impact of tagging, only adult and sub-adult whales not accompanied by calves were tagged and a maximum of three tagging attempts (approaches of the boat within 100 m of the whales) were performed on a group of whales in any given day.

**Table 1:** Dive and foraging statistics.

Whale	Date	Tag duration	FD/FD bot	FD duration (mean, range)	FD max. depth (mean, range)	SOC depth (mean, range)	Search (mean, range)	# Buzz (mean-range)
MdH1	2008-05-16	18.4	7/7	48(33–65)	911(491–1330)	448(179–873)	28(21–38)	35(18–49)
	2005-10-21	4.1	3/2	50(48–51)	671(597–790)	475(457–492)	22(22–22)	24(20–28)
	2003-10-11	12.5	5/3	51 (40–57)	616(616–616)	414(183–566)	26(18–31)	26(12–44)
MdH15	2003-10-25	2.6	2/2	47 (45–48)	774(732–815)	426(416–434)	25(23–27)	23(20–27)
MdH22	2008-10-15	18.0	7/2	44(23–57)	710(472–963)	340(193–560)	23(9–32)	21(4–34)
	2005-10-21	2.8	1/0	47(47–47)	616(616–616)	520(520–520)	21(21–21)	18(18–18)
	2004-10-13	9.5	4/3	44(34–55)	1003(715–1311)	473(448–499)	28(23–33)	32(25–37)
MdH6	2008-05-15	2.0	2/2	48(44–52)	781(779–784)	389(326–454)	24(20–27)	23(19–27)
	2005-10-04	6.9	3/3	57(51–62)	914(869–953)	518(513–524)	25(22–29)	29(23–37)
MdH43	2005-10-12	8.6	4/4	45(39–52)	833(674–1011)	505(482–540)	25(25–26)	43(37–53)
MdH74	2008-05-21	1.6	1/1	47(47–47)	807(807–807)	419(419–419)	20(20–20)	11(11–11)
MdHC1	2008-05-27	6.2	2/2	58(52–64)	932 (840–1024)	461(435–486)	27(24–31)	34(32–37)
MdHX33	2010-05-26	2.9	1/1	48(48–48)	925(925–925)	503(503–503)	22(22–22)	34(34–34)
MdH86	2010-06-10	15.3	8/8	41(31–51)	834(784–915)	353(169–517)	22(16–29)	18(12–29)

Summary of statistics for 50 foraging dives from 14 tag deployments on 9 Blainville's beaked whales off El Hierro. Values in the last 5 columns are means over each tag deployment with the range given in parentheses. Whale: database code of the tagged whale (www.cetabase.info); Date: year, month and day of tagging; Tag duration: tag recording duration in hours. FD: number of complete foraging dives; FD bot: number of complete foraging dives containing echoes from the sea-floor; FD duration: length of foraging dives in minutes. SOC depth: depth in meters of start of regular clicking; Search: time elapsed between start and end of clicking in each dive; # Buzz: number of buzzes emitted per dive.

### Dive cycle

Tag acoustic recordings were evaluated by examining consecutive spectrograms (512 sample Hann window, 1024 bin FFT) of 20 s of data to locate buzzes, and the beginning and end of the vocal phase in each dive. Buzzes are considered indicative of prey capture attempts [31], [39] while the beginning and end of vocalizations (clicks) in a dive are taken as indicating the duration of prey search by echolocation [33], [34]. A supervised click detector (a band-pass energy detector with a user-selected threshold) was used to identify individual clicks for later echo analysis. Based on the sound events and dive profiles, we divided the dive cycle into three phases: (i) *transport*: the time elapsed from leaving the surface to the start of echolocation clicking (SOC) plus the time elapsed from the end of clicking (EOC) to re-gaining the surface; (ii) *search*: time elapsed from SOC to EOC in a foraging dive, i.e., the time spent echolocating; (iii) *inter foraging dive interval* (IFDI): time spent at the surface or in silent shallow dives between deep foraging dives. Only full dive cycles (i.e., a foraging dive+complete IFDI), and therefore only those tag records with at least one full dive cycle, were examined for time allocation in these dive phases.

### Foraging activity

To test for circadian changes in foraging behavior, we performed a day/night comparison of: i) the depth of the start of clicking and the first buzz in dives; ii) the number of buzzes per dive; iii) the maximum buzz depth in each dive, and (iv) the proportion of time spent in search and transport for all individuals. A dive was considered to be performed during the day or night if the SOC occurred before or after local sunset. Comparisons were made with nested ANOVA using day-night as the main grouping factor and individual as the sub-grouping factor. The relative importance of the main and sub-grouping factors was estimated using partial-eta-squared coefficients (pη^2^) [40].

To test for a circadian change in the depth range over which foraging took place, depth distributions in 50 m depth bins were computed for the search time, buzz count, and buzz rate (i.e., the number of buzzes in each depth bin divided by the time spent in that depth bin) in 4 tag deployments on 3 individuals that spanned day and night. These distributions indicate, respectively, the depths at which whales search for and encounter prey, and the rate at which they find prey as a function of depth. Individual depth distributions in day and night dives were compared using a Kolmogorov-Smirnov test.

### Echoes from the sea-floor and organisms

**Figure 1:**
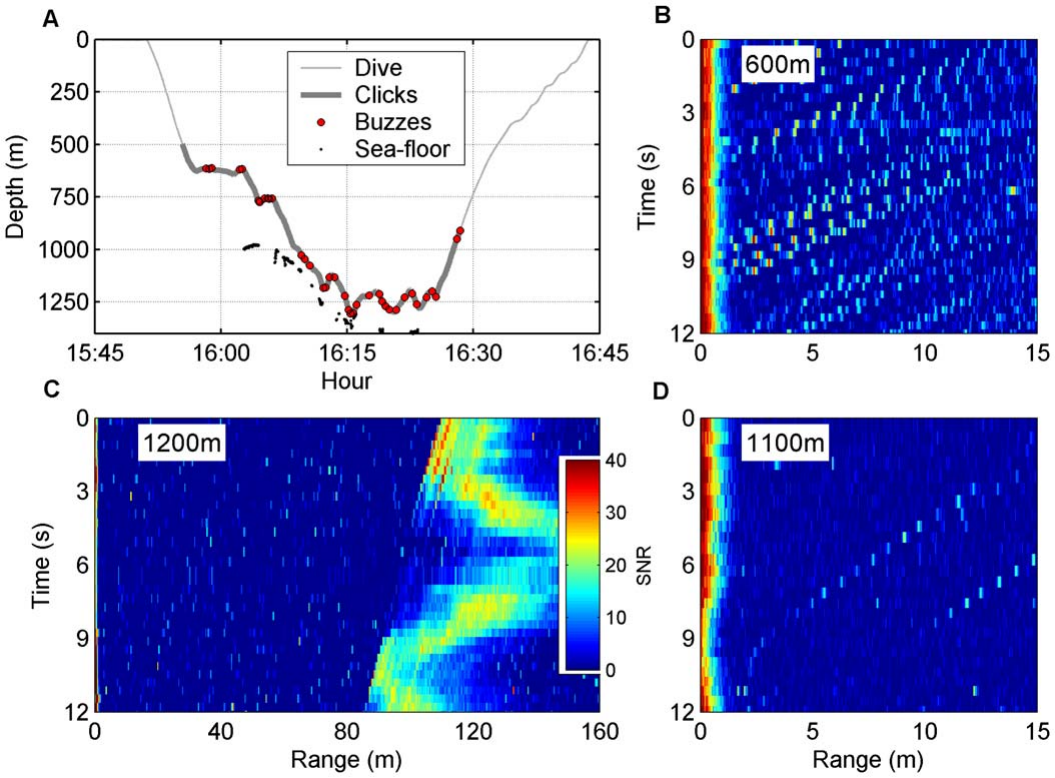
Foraging dive profile and echograms showing sea-floor and organisms ensonified by the whale. (A) Dive profile of a female Blainville's beaked whale (MdH22 tagged on 10/03/04) showing the vocal phase of the dive (thick grey line), the location of the buzzes (black circles) and the seafloor (red dots). (B, D) Echograms with a range span of 15 m indicating the distance from the whale to echoic organisms on consecutive clicks. Echoes appear as dots coloured by the signal-to-noise ratio of the echo. In these examples, the echo counts are 198 echoes/60 clicks (B) and 6 echoes/35 clicks (D). The red thick lines on the left of the plots are the clicks emitted by the whale. (C) Echogram with a range span of 150 m showing reverberant reflections off the sea-floor which was 90–120 m below the whale.

All tags recorded echoes from both the sea-floor and from organisms near the tagged whale ensonified by clicks from the whale. Echoes were identified using echograms generated as a stack plot of the envelope of high-pass filtered sound segments following each out-going click (*sensu* [31]). The cut-off frequency of the filter was set to the lower −10 dB frequency of Blainville's beaked whale frequency-modulated (FM) clicks (27 kHz) [38]. Sound segments of 1 s duration were used to detect sea-floor echoes at distances of up to 750 m from the whale. Echoes from the sea-floor appeared in echograms as light-shaded areas (Fig. 1C) with an abrupt onset time corresponding to the two way travel time (TWT) from the whale to the closest sea-floor surface. The TWT was estimated using a supervised edge detector (resolution<500 µs) and converted to the altitude of the whale above the sea-floor (whale-altitude here on) by multiplying the TWT by one half of the path-integrated sound speed. The TWT to range conversion was iterated several times from a fixed starting sound speed estimate (1500 m/s) to resolve path length and path-integrated sound speed in tandem. The sound speed profile required by this algorithm was measured with a CTD cast to a depth of 1300 m in the same area and extrapolated to deeper depths using a constant temperature and salinity assumption. The sea-floor depth was estimated by adding the whale-altitude to the depth of the whale (whale-depth here on) as recorded by the tag pressure sensor. The low source level of buzz clicks (some 20 dB lower than FM clicks) [32] precluded the detection of sea-floor echoes during buzzes. Thus, the whale-altitude during buzzes was estimated using sea-floor depths acquired up to 60 s before or after each buzz, corrected by the whale-depth during the buzz. The mean change in sea-floor depth recorded over 60 s intervals outside of buzzes during the vocal search phase was 9 m, indicating the likely order of error incurred by this approximation.

Echoes from organisms in the water column were recorded at ranges of more than 20 m from the whale and varied in density of occurrence from a few discrete echoes to nearly continuous back-scatter (Fig. 1B,D). Manual identification of sequences of echoes from distinct organisms has been attempted previously [32], [41] but this is unreliable when echo density is high. To quantify the abundance of echoes as a function of depth over a range of echo densities we used an automatic echo counting method. The RMS sound pressure level was computed for ten 1 ms long samples of filtered sound (6-pole Butterworth band-pass filter with 25–50 kHz cut-off frequencies) taken from the 5th to the 15th ms after each click produced by the whales (corresponding to echoic targets 3.75 to 11.25 meters from the whale). The RMS level in each of the 10 sound samples was compared against the RMS level of a 10 ms sound sample taken just before the same click, with the same filter settings, representing a measure of the instantaneous ambient sound level at the tag. This comparison provided an estimate of the signal-to-noise ratio (SNR) in each post-click sound sample and samples with SNR ≥6 dB were considered to include an echo. The number of these samples containing echoes was averaged over all clicks produced by all whales in 50 m depth bins to produce a nominal measure of echoes per click in each depth bin. Data from two tags (summing 12 dives) were excluded from this analysis because echoes in these tags were masked by high flow noise, possibly due to a caudal tag placement.

To validate the automatic echo counting method, results were compared to manual echo counts obtained by inspection of echograms on a sub-set of the data. Results from the two methods were correlated with a slope of 0.6 (Spearman correlation ρ = 0.80, n = 953 clicks, 422 echoes identified manually and 296 echoes gathered with the automatic tool). The automatic method gave consistently lower counts than the manual method due to the relatively conservative detection threshold and averaging window (6 dB and 1 ms, respectively) in the former. The number of echoes detected from a series of clicks is dependent on the number and target strength of organisms ensonified, and also the number of consecutive clicks that each organism remains in the echolocation beam. This last factor depends on the types of organisms ensonified and also on the whale's movements which may vary throughout a dive. As a result, the echo count described here should be considered as a relative measure of organismal density rather than a direct estimate of the number of organisms that the whale encounters.

### Oceanographic and hydroacoustic data

Sound speed and oxygen concentration depth profiles were gathered with a conductivity, temperature and depth recorder (CTD, RBR XR-620) lowered at a rate of 60 m/min to 1000 m depth. A hydroacoustic survey was performed in June 2009 using an uncalibrated SIMRAD EK-60 splitbeam echosounder operating at 38 kHz with a beamwidth of 7.2°. Data were collected using 2 kW pulses of 1024 µs duration and sampling interval 0.196 m. Background noise (approx. −129 dB re m^−1^) was automatically estimated using the minimum sample power in bins of 20 pulses horizontal and 10 m range following De Robertis & Higginbottom [42]. The mean volume-backscatter strength (dB re m^−1^), a logarithmic measure of volume backscattering [43], was estimated from 0 to 1000 m depth and visualized with the aid of echograms after noise correction. Hydroacoustic transects, each covering 3680 m (2 nm), were performed day and night between the 1000–2000 m isobaths some 3 km from the shore. Both CTD and hydroacoustic data were gathered in the core area of distribution of Blainville's beaked whales off El Hierro.

## Results

**Figure 2:**
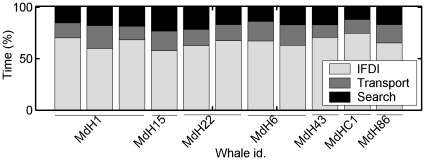
Time budget. Time budget of seven whales in 11 tag deployments with at least two complete foraging dives. The bars represent the proportion of time invested in: inter foraging dive intervals (IFDI, light grey), silent transport to and from foraging depths within deep foraging dives (dark grey) and searching (i.e., echolocating) for prey (black).

A total of 111.4 hours of combined acoustic and movement data were gathered from 14 tag deployments, including 50 complete foraging dives and 33 dive cycles (Table 1). Tagged Blainville's beaked whales performed long and deep foraging dives with a mean duration of 48 min (23–65 min) and a mean maximum dive depth of 833 m (472–1330 m) (values are given as the *mean* with the *range* in parentheses). Whales started echolocating at a mean depth of 425 m (169–873 m) after a silent descent lasting on average 4 min (1–11 min). Echolocation stopped at a mean depth of 712 m (273–1027 m) and was followed by a silent ascent lasting on average 19 min (9–35 min). The sum of the silent descent and ascent phases resulted in a mean transport phase duration of 23 min (12–33 min). The search phase lasted on average 24 min (9–38 min) (Fig. 2), with whales producing an average of 27 buzzes per dive (4–53 buzzes).

Between consecutive deep dives, whales spent a mean of 92 min (4–188 min) performing a series of shallow and silent dives not related to foraging [34]. Thus, tagged Blainville's beaked whales devoted 18% of their time to transport within foraging dives, 18% in search and acquisition of prey, and 64% to non-vocal shallow diving between deep dives (Fig. 2). Within the searching phase, whales spent on average only 2 min (0.6–3.1 min) echolocating before making the first prey capture attempt in each dive.

### Day/night foraging preferences

**Figure 3:**
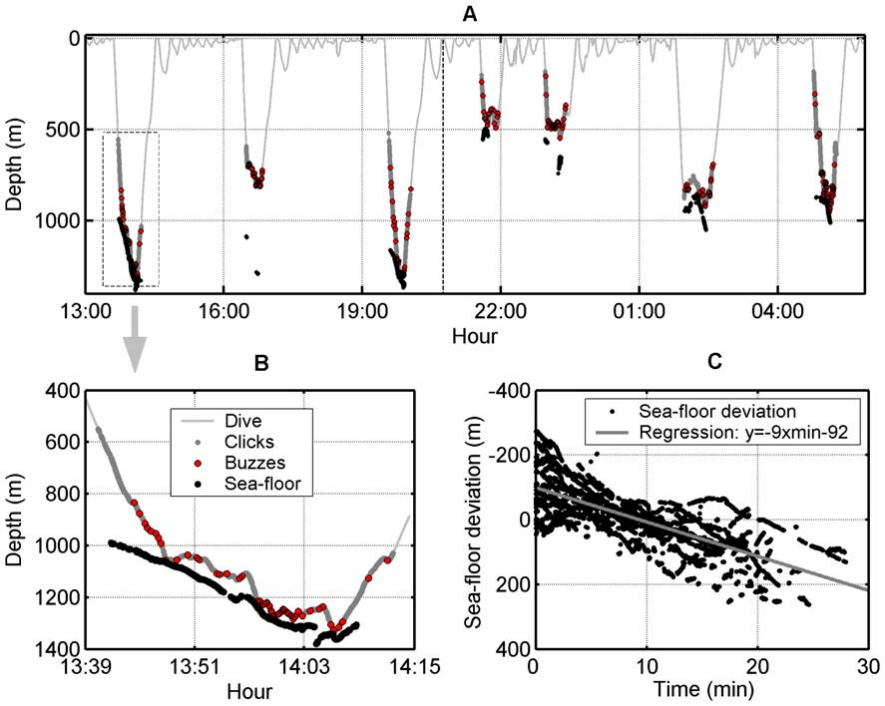
Whale foraging activity in relation to the sea-floor. (A) Dive profile of a male Blainville's beaked whale (MdH1 tagged on 16/05/08). Thick grey lines indicate the vocal phase of the dives (echolocation) and grey circles represent foraging attempts (buzzes). Black dots show the location of the sea-floor and the vertical dashed line at 20:45 h indicates sunset. The whale approached the sea-floor in most dives despite diving to a wide range of depths. (B) Detailed view of part of the first dive when the whale follows the sea-floor contour to forage. (C) Deviation of the sea-floor depth, in the directions taken by tagged whales, as a function of dive time. The sea-floor deviation is the instantaneous sea-floor depth minus the mean sea-floor depth over each dive. The negative slope of the regression line indicates a tendency for whales to forage down-slope.

**Figure 4:**
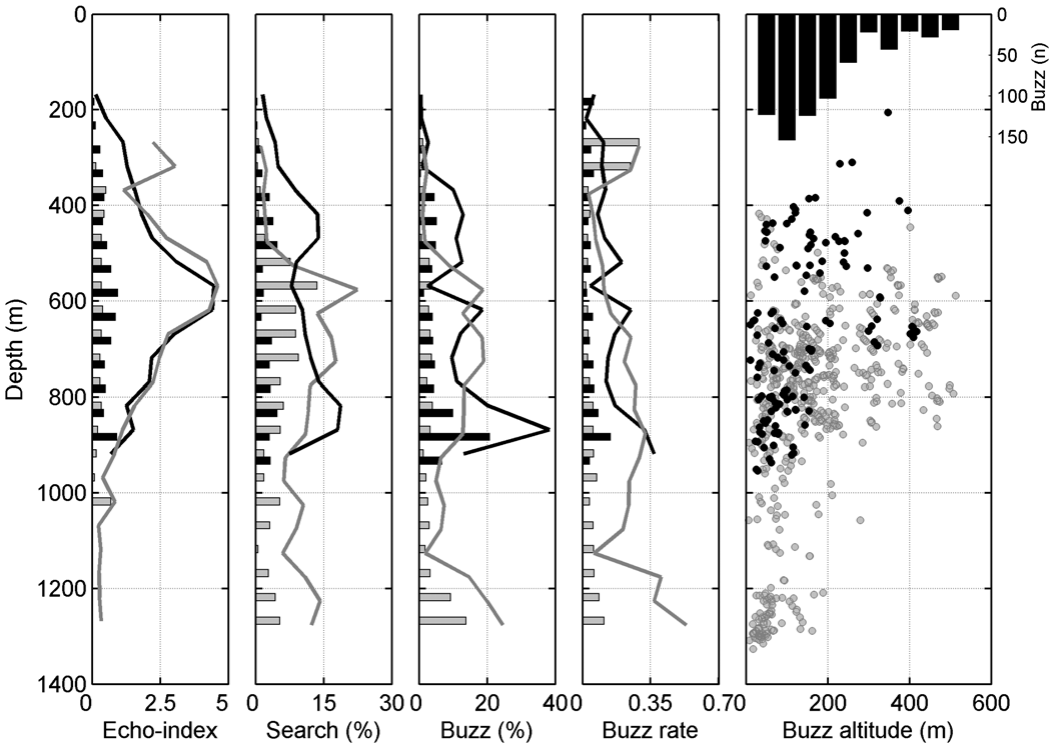
Day and night depth profiles of echo and foraging parameters. (A) Echo-index, indicative of the relative density of echoic organisms insonified, (B) proportion of time spent echolocating per depth bin, (C) proportion of buzzes produced per depth bin, (D) buzzes per minute spent in each depth bin. Lines and bars correspond to the mean and standard error, respectively, of the above parameters for day (grey) and night (black) dives. These plots represent pooled data for all dives from all tag deployments in 50 m depth bins except the echo-index, which was not possible to quantify for two tags (6 dives) due to high flow-noise levels. (E) whale depth and altitude above the sea-floor for each buzz recorded within 60 s of a sea-floor echo (black and grey dots for night and day time buzzes, respectively) and histogram of whale-altitude in 50 m bins for those buzzes (top black bars).

**Figure 5:**
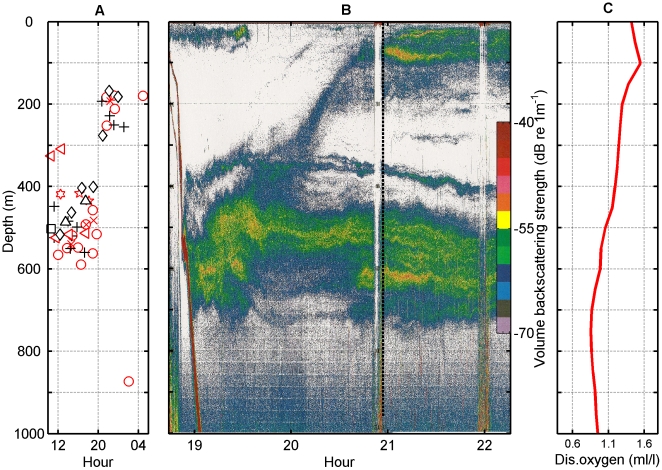
Whale start of clicking in relation to biological and physical parameters of the study area. (A) Depth at which tagged whales (n = 9 individuals, identified with different symbols) start echolocating in 50 foraging dives performed at different hours of the day. (B) Echosounder profile showing the depth distribution of the biomass in the study area off El Hierro during the day-night transition. The colours indicate echo intensity in dB re 1 m^−1^. The vertical dashed line indicates the time of sunset and the red areas correspond to echoes from the sea-floor. The white bands result from outages in echo reception due to air bubbles produced by manoeuvres of the boat. The circadian vertical migration of the deep scattering layer is evident. (C) Profile of the dissolved oxygen concentration (ml/l) in the same waters.

Diel differences were tested in 11 tag deployments on 9 individuals that contained at least two complete dives. These recordings comprise in total 73 hours and 46 dives, (15 hours and 12 dives of which occurred at night-time). Tagged whales started clicking (i.e., searching for prey) and emitting buzzes (attempting to capture prey) at significantly shallower depths at night than during the day (Figs. 3A, 4E and 5A). The mean SOC depth was 479 m (276–589) and 258 m (168–873) for day and night, respectively (nested ANOVA, p = 0.001, pç^2^ = 0.55 for diel factors and p = 0.42, pç^2^ = 0.37 for individual factors) and mean depth of the first buzz was 755 m (563–1027) and 572 m (273–904) for day and night, respectively (nested ANOVA, p = 0.005, pç^2^ = 0.57 for diel factors and p = 0.08, pç^2^ = 0.50 for individual factors). Because both the depth at which whales started clicking and the depth of the first buzz changed by about the same amount from day to night, there was little diel variation in the time spent searching for prey before the first buzz, 2.1 min (0.04–4.5) during the day and 1.9 min (0.3–4.9) at night, (Ranksum, p = 0.53, n = 46 dives).

Although whales started to forage shallower at night, there was no detectable change in the depth distribution of search time or buzz rate from day to night in the 3 individuals that could be tested. These whales performed both day and night-time dives allowing for the comparison of these parameters in a tag-by-tag basis (15 and 12 dives in day and night-time respectively). The depth distribution of buzzes only showed significant circadian variations in one of the three individuals (Kolmogorov-Smirnov, n = 15 depth bins of 50 m, p = 0.01 for that deployment). There was no evidence for circadian changes either in the maximum depth of buzzes, the number of buzzes per dive, or the time allocated to transport and foraging within dives. In summary, whales searched and found prey over a greater depth range, starting at shallower depths at night-time than during the day, but they made use of deep foraging resources both day and night.

### Altitude of foraging

Sea-floor echoes were found in 13 of the 14 tag deployments (the only tag without sea-floor echoes recorded a single foraging dive) and in 40 of the 50 foraging dives (Table 1, Fig. 3A). Over all tags, the sea-floor depth, as calculated from echoes, varied between 414 and 1425 m with a median of 885 m. Whale-altitude above the sea-floor varied from 535 to as little as 5 m (i.e., about a body length) with a median of 127 m. Using a 60 s extrapolation interval, whale-altitude and bottom depth could be estimated for 64% of the echolocation time. Sea-floor echoes will become weaker and harder to detect as the whale-altitude increases and so some intervals without echoes may occur in water that is too deep to register echoes. However, sea-floor echoes also should be detected most readily when whales are pointing downwards and so more likely to ensonify the sea-floor with their narrow forward-directed sonar beam. That orientation is a key determinant of whether a bottom echo is detected was confirmed by comparing the distribution of pitch angles of whales when echoes were recorded against the overall pitch angle distribution of whales while clicking (Wilcoxon rank-sum, p≪0.001 for equal median pitch angles, n = 11228 clicks with bottom echoes compared with 55155 clicks with and without echoes). Some 70% of clicks with sea-floor echoes were obtained at pitch angles below 0° (a downwards pointing whale has a negative pitch angle), while the overall pitch distribution during foraging was roughly symmetric around 0°.

When the sea-floor depth could be tracked reliably, whales often seemed to follow a downward sloping sea-floor while they foraged (Fig. 3). To test if this was a stereotyped behavior, we estimated the slope of the sea-floor along the course taken by the whales, in terms of meters of depth change per minute (converting this to a more conventional slope in meters per meter requires the whale's speed which was not available). Pooling all dives with sea-floor echoes and removing the mean sea-floor depth in each dive, a mean sea-floor slope of −9 m/min was found (Spearman correlation between time and bottom depth, ρ = 0.68, p≪0.001, 40 dives with at least 5 depths/dive, n = 6142 sea-floor depths). Regressing individual dives, all 40 dives with sea-floor echoes had a negative bottom slope, confirming that tagged whales routinely swam down-slope when foraging.

Discontinuous detection of sea-floor echoes resulted in whale-altitude estimates for only 535 of 974 buzzes (i.e., 55%), spanning 40 of 50 dives. Buzzes with measurable altitude were performed at a median altitude above the sea-floor of 139 m (5–507 m) and mean depth of 815 m (309–1326 m). Two general trends are evident in the whale altitude data (Fig. 4E): at whale-depths of up to 900 m, encompassing a majority of the buzzes, whales fed at a range of altitudes above the sea-floor but mostly at altitudes <250 m. At deeper foraging depths, feeding took place almost exclusively within 200 m of the sea-floor. There is certainly little evidence in Fig. 4E for pelagic foraging deeper than about 900 m. However, pelagic foraging may occur in shallower parts of dives and involve some of the buzzes for which sea-floor echoes were not obtained. Buzzes without sea-floor echoes tended to be shallower (mean of 696 m, range 359–1288 m) than buzzes with echoes, but were not necessarily made with an upward pitch angle (mean pitch angle in the surrounding 60 s of each buzz was 1°), suggesting that the sea-floor was out of detection range in many of these buzzes.

Following the traditional definition of the benthic boundary layer (BBL) extending 200 m above the sea-floor [9], 78% (n = 420) of buzzes with measurable altitude occurred within the BBL, indicating benthopelagic foraging. These BBL buzzes were produced at whale-depths from 385 to 1326 m, suggesting that whales were targeting the BBL in these buzzes rather than a specific water depth. This assertion was supported by comparing the variability in whale-altitude and whale-depth for all buzzes with measurable altitude. The interquartile range (IQR) for altitude (125 m) <IQR for depth (197 m), rank-sum p≪0.001 on n = 1000 bootstrap samples of IQR. Of the BBL buzzes, some 57% (n = 240) were recorded within 100 m of the sea-floor while nearly 18% (n = 74) were performed at more than 150 m altitude, close to the nominal upper edge of the BBL, making the distinction between bentho- and meso-pelagic foraging somewhat diffuse.

### Echo index and foraging activity

Echoes from organisms in the water column, as detected in tag recordings, were most abundant at depths between 500 and 750 m both during day and night-time (Fig. 4A; day = 12 tags, 31 dives; night = 3 tags, 6 dives). Echo counts per depth-bin were lower at night than in day-time, at least at depths between 350 and 900 m where both day and night data are available (Wilcoxon signed-rank, p≪0.001 for median equal to 0 , n = 13 depth bins) but echo counts at <500 m depth during the day and <200 m depth at night may be unreliable because they are based on only a few clicks. The depth distribution of echoes during the day matched fairly closely to two different proxies of foraging effort: the proportion of time spent searching (i.e., making echolocation clicks) and the proportion of buzzes (Spearman correlation ρ = 0.49, p = 0.01, n = 20 for search time and ρ = 0.46, p = 0.02, n = 20 for proportion of buzzes) (Fig. 4A–C). The same proxies of foraging effort were less clearly correlated with echo counts at night-time (Spearman correlation, ρ = 0.42, p = 0.06, n = 15 for search time and ρ = 0.32, p = 0.12, n = 15 for proportion of buzzes) with the highest echo counts being recorded some 150 m below the depth of greatest foraging effort at night. Thus, foraging during the day concentrates at the depth of higher echo counts and below it, in deeper waters, while most night-time foraging occurred above and below this depth layer. The number of buzzes and the time spent in each depth bin were closely correlated with each other, day and night (Spearman correlation ρ = 0.87, p<0.001, n = 16 for night dives and ρ = 0.97, p<0.001 n = 21 for day dives), resulting in a relatively constant buzz rate (buzzes per minute per depth bin) throughout the water column (Fig. 4D).

### Oceanographic and hydroacoustic data

CTD casts showed a decreasing oxygen concentration with depth reaching a minimum of 0.85 ml/l at 751 m, roughly one half of the surface concentration. In deeper waters and up to the maximum recording depth (1000 m), oxygen concentration increased gradually up to 0.94 ml/l. Echosounder data showed a stable DSL between 500–750 m depth both during day and night (Fig. 5B), broadly comparable to the on-animal echo counts (Fig. 4A). At sunset, a portion of the DSL organisms migrated to epipelagic waters to form a second thinner but equally dense layer of organisms, extending from the surface to 200 m depth. This layer was not detected in the on-animal echo counts because echolocation clicks were rarely produced <200 m.

## Discussion

Foraging decisions of air-breathing marine predators revolve around locating sufficiently rewarding food patches in a limited dive time, while maximizing the ratio between energy acquired and oxygen spent [18], [28]. Despite the challenges involved in foraging at depth for marine mammals, more than 20 species have evolved to access mesopelagic depths as top predators in a hostile world of high pressure and darkness. However, little is known about the forces driving niche segregation and habitat selection in deep-diving marine mammals, even though deep waters are one of the largest ecosystems on the planet, and consequently offer a great variety of potential niches and habitats. Foraging resources in the oceans are not homogeneously distributed and deep-diving species must balance the transport costs to reach preferred foraging layers against the caloric value, abundance and the ease with which prey can be caught in these layers [21]. Solutions to this problem vary according to the diving and hunting capabilities of each species [45] but also according to the instantaneous availability of prey, as sensed by the animal [26]. Here we report for the first time the fine-scale meso- and benthopelagic habitat use of a deep diving marine mammal using unique information about the location of biomass in the water column, and the location of the sea-floor, as sensed by the animal itself using echolocation.

### Dive cycle & time budget

Blainville's beaked whales perform long, deep foraging dives which have been described as extreme for the size of this 4–5 m whale [34], [44]. Our results are consistent with previous reports based on a sub-set (28 hours) of the current data-set [34] or obtained using tags without acoustic sensing [44], confirming the extreme diving behavior of this species with dives on average up to 833 m and 48 min duration. Probably as a consequence of performing such protracted dives, which likely involve considerable lactate build up [34], the whales only spend about one third of their time performing foraging dives, of which just one half is spent searching for, and capturing prey. Silent transport between the surface and the foraging layers account for the other half of the duration of each foraging dive.

Compared to sperm whales [33], another echolocating predator foraging at similar depths, Blainville's beaked whales thus spend notably less time per dive searching for prey (70–90% of dive time for sperm whales vs. 50% for beaked whales). This is mainly because beaked whales perform long and low-angle silent ascents from foraging dives [34] while sperm whales ascend vertically to the surface from their foraging depths [33]. Adding the long inter-foraging-dive intervals, Blainville's beaked whales spend less than a third of the time that sperm whales do searching for prey (68% for sperm whales vs. 18% for beaked whales). This apparent higher foraging efficiency of beaked whales probably reflects differences in foraging requirements between these very different sized animals, as well as the greater diving capacity of sperm whales with a body mass that allows them to extend the foraging time at depth. Beaked whales seem to have adapted to exploit a reliable niche at the cost of performing protracted dive lengths for their size and thus requiring extended resting periods between dives [34]. The relative short foraging periods of the Blainville's beaked whales and the observation that they only initiate searching when already deep in the descent and yet encounter prey suitable for capture within 2 min of the start of echolocation, further supports that these whales are accessing prey in reliable vertical strata, and are likely using cues other than echolocation, such as depth, to guide their biosonar based search for prey. Moreover, these prey resources are sufficiently dense to feed the animals in what is effectively four hours of hunting per day. However reliance on such predictable dense resources may tie Blainville’s beaked whales to specific habitats where these are available. In the following we explore what the tag-recorded echolocation data reveal about the location of organisms and the foraging choices of Blainville's beaked whales.

### Diel foraging strategy

Echoes from the sea-floor were detected during most foraging dives and these indicate that Blainville's beaked whales feed both in mesopelagic waters and as close to the sea floor as 5 m. This foraging behavior seems to exploit two stable concentrations of biomass. Most mesopelagic prey capture attempts are performed when the whales are swimming at relatively shallow depths (500 to 850 m) broadly coinciding with the lower part of the DSL (Fig. 4A,E). In contrast, whales approach the sea-floor to forage over a wider range of depths, and seem to target exclusively benthopelagic organisms when foraging deeper than 900 m. The steep coastal bathymetry of El Hierro offers a variety of foraging environments on a small spatial scale, with the 1000 m and 2000 m isobaths as close as 1.3 km. In this topography, Blainville's beaked whales can switch between meso- and bentho-pelagic foraging in the same dive (e.g., dive 3, Fig. 3A). Foraging choices are probably guided by the type and abundance of prey encountered in each habitat on a dive-by-dive basis, as has been suggested for similar foraging transitions in other marine mammals [22].

The foraging altitude of tagged whales is estimated here using a method that is reliable in steep bathymetry and does not require horizontal localization of animals or accurate bathymetric charts. However, altitude cannot be estimated for about half of the prey capture attempts due to either an unfavourable orientation of the animal or too greater distance to the sea-floor. This may lead to an underestimation of the proportion of mesopelagic foraging in our data. But even if all of the buzzes without sea-floor echoes are mesopelagic, at least 43% of buzzes (420/974) are within the BBL, and many dives contain buzzes in each category, confirming the foraging importance of both domains for Blainville's beaked whales.

Most mesopelagic buzzes during the day occur at and below the peak of the echo-index, indicating that whales are targeting the lower levels of the DSL (Fig. 4E and Fig. 5B), possibly foraging on deeper-living mesopelagic species that are themselves predators of DSL organisms [12]. During the night, whales start searching for food at shallower depths than in daytime (Fig. 5A) and nocturnal foraging peaked at some 150 m above the main layer of the DSL, suggesting some adaptation of foraging effort to target migrating DSL species (Fig. 4E and Fig. 5B). However, the whales also perform deep dives at night-time to forage on non-migratory or partial migratory organisms of the DSL and on bentho-pelagic prey (Fig. 4E). As a result, the maximum depth and time budget of dives, as well as the overall depth distribution of foraging effort change little from day to night. At first glance, this behavior seems to waste energy in transport without increasing the probability of finding prey, since whales only adapt partially to the depth distribution of the biomass in the water column. However, a closer look at the search behavior of Blainville's beaked whales suggests that they prioritize certain types of prey rather than absolute biomass availability in the water column.

### Prey preferences

Net energy intake for a predator is determined not only by acquisition rate but also by the cost of capturing prey and its caloric content [1]. Caloric contents and locomotory capacities are usually lower in demersal as compared to pelagic species, and tend to decrease with increasing water depths both within the ocean at large and within the DSL [14], [46]. The foraging effort of Blainville's beaked whales is concentrated, at least during the day, in the lower levels of the DSL or just below it, coinciding well with the oxygen minimum layer (OML) in the study area (Fig. 5). The OML is a region usually dominated by organisms with low metabolic rates as an adaptation to the low oxygen concentration in the water [14], [47] and hence likely with little movement capacity relative to a breath-holding mammalian predator. In contrast, organisms at the medium and upper levels of the DSL have generally higher metabolic rates, representing more active species, especially those that migrate to epipelagic waters at night, and may be better able to perform sustained escape responses to avoid capture [11], [48]. Migratory organisms tend to be “dormant” during the day but become very active at night, mainly those migrating to epipelagic waters [12]. The potential avoidance capability of prey probably explain why Blainville's beaked whales do not forage shallower than 200 m depth, despite the near-surface biomass concentration observed in ship-borne echosounder data at night (Fig. 5). Blainville's beaked whales do not seem to chase their prey over long distances and tend to approach them at slow speeds [32], which supports the idea that they might be targeting prey that are individually low cost to hunt even though this involves an increased cost of transport to reach them at deeper depths. This focus on slow prey is rewarded by the capture of some 30 prey per dive, albeit likely with a low individual caloric content but sufficient to fulfil the energetic requirements of the whales in just four hours of hunting per day.

### Search behaviour and habitat use

We have shown that Blainville's beaked whales switch between meso and bentho-pelagic habitats with foraging decisions being made on a dive-by-dive basis. Whales start echolocating above the DSL and sometimes continue to forage in the mesopelagic domain, probably exploiting more lethargic prey around the OML during the day and non- or partially-migratory DSL organisms at night. In other dives, whales descend below the DSL and approach the sea-floor to search for prey in a down-slope direction. In both cases, the whales start searching for meso- or bentho-pelagic species at relatively shallow depths, and perhaps only commit to deeper bentho-pelagic foraging if shallower prey are not found. The bentho-pelagic habitat in steep slopes around oceanic islands and sea-mounts is often enriched, as these slopes function as ecotones where the pelagic and benthic domains overlap. High concentrations of organisms may be found close to the sea-floor in this habitat as mesopelagic fauna impinge on the slopes, mixing with, and providing additional foraging resources for, demersal species [49]. There are no data on the deep-water productivity of El Hierro but the steep topography of the island [50] suggests that such local-scale enrichment phenomena may occur there. This would explain the presence of a year-round population of Blainville's beaked whales in an area with oligotrophic surface waters [51] that would seem unlikely to sustain a group of large endothermic predators.

The foraging behavior described here may be specific to the resident population of Blainville's beaked whales in El Hierro, adapted to the local topography of the island. However, this species has been reported to distribute over continental slope areas and around oceanic islands in other sub-tropical regions [44], [52]. In the three coastal areas of the world where resident populations of any of the 21 species of beaked whales have been found, Blainville's beaked whales are the species that tends to be found in shallower waters nearer the shore [52], [53]. The foraging behavior quantified here, and the apparent preference of this species for steep bathymetry, may then help explain its overall distribution. It may be germaine to consider this apparent habitat preference of Blainville's beaked whales when planning activities, such as naval exercises or seismic prospections, that have been related to mass strandings of beaked whales [54], [55]. However, the cost and complexity of oceanic studies of beaked whales leads to a strong bias in effort towards coastal populations that are relatively easy to access. Given the large number of beaked whale species and their occurrence in all oceans they almost certainly occupy habitats beyond steep slopes. This is important to keep in mind when extrapolating results from coastal studies.

### Conclusions

We have shown that Blainville's beaked whales spend only four hours per day hunting for food, with such a short foraging time likely resulting from long transport times to foraging depths and long recovery times between deep foraging dives. This necessitates a stable and abundant prey resource that can be located reliably in an extensive 3-dimensional world of darkness. The steep sloping terrains in locations where Blainville's beaked whales are often found may offer access to resources associated with both the DSL and the BBL over a small spatial scale. Echolocating whales can glean both biotic and abiotic cues to aid the efficient location of these resources from biosonar echoes. The enigma of why Blainville's beaked whale abundance is apparently strongly linked to a dense DSL [35], even though they seem to forage outside the DSL, may then be explained by the observation here that whales target prey in the oxygen minimum layer associated with, but deeper than the bulk of the DSL. Thus, by inhabiting steep undersea slopes, Blainville's beaked whales can target a stable and abundant resource of mixed meso and benthopelagic prey using biosonar-derived landmarks.
