# Correction: Following a Foraging Fish-Finder: Diel Habitat Use of Blainville's Beaked Whales Revealed by Echolocation

**DOI:** 10.1371/annotation/c165133f-34fd-4cca-b84f-fc30de881332

**Published:** 2012-01-04

**Authors:** Patricia Arranz, Natacha Aguilar de Soto, Peter T. Madsen, Alberto Brito, Fernando Bordes, Mark P. Johnson

There is an error in the given name of the second author in the citation. The correct citation is: Arranz P, Aguilar de Soto N, Madsen PT, Brito A, Bordes F, et al. (2011) Following a Foraging Fish-Finder: Diel Habitat Use of Blainville's Beaked Whales Revealed by Echolocation. PLoS ONE 6(12): e28353. doi:10.1371/journal.pone.0028353 

