# Correction: Following a Foraging Fish-Finder: Diel Habitat Use of Blainville's Beaked Whales Revealed by Echolocation

**DOI:** 10.1371/annotation/49be01ca-99fb-40f2-bb9c-9720c87be632

**Published:** 2012-03-29

**Authors:** Patricia Arranz, Natacha Aguilar de Soto, Peter T. Madsen, Alberto Brito, Fernando Bordes, Mark P. Johnson

There was an error in the scale of the x-axis in Figure 5C. The correct Figure 5 can be viewed here: 

**Figure:**
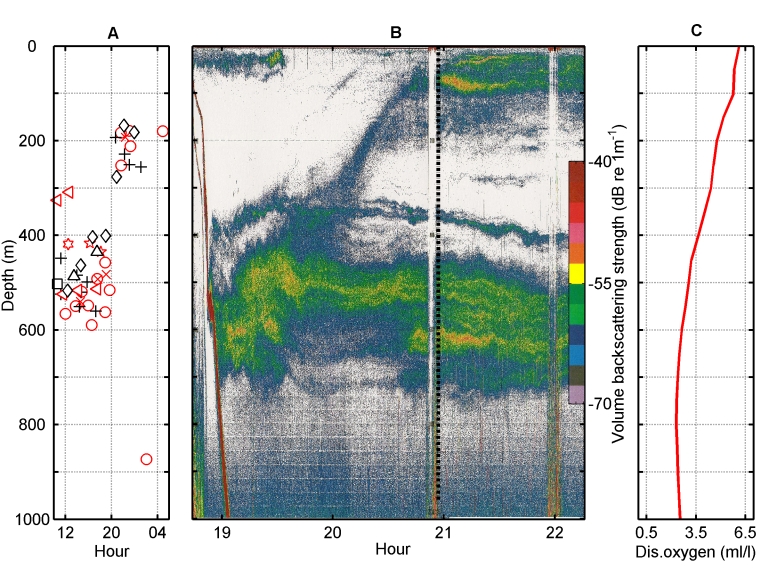


.

